# Supplementary material for: MRI-Based Assessment of Brain Tumor Hypoxia: Correlation with Histology
Source: Cancers (Basel). 2023 Dec 27;16(1):138. doi: 10.3390/cancers16010138 (PMC10778427; doi:10.3390/cancers16010138)
Supplement: Supplementary file 1 [file cancers-16-00138-s001.zip › cancers-2720704-supplementary.pdf]

Supplementary Table S1: Median measurements of R_2', DBV, OEF, CBV and vessel size for ten patients across various VOIs. Values in parentheses indicate the interquartile range. Instances marked 'N/A' highlight VOIs where the sqBOLD model isn't applicable, notably in the edema sections of patient-09 and patient-04 (diagnosed with brain metastasis), which were excluded due to more than 80% failure voxels.

Patient	VOI	R_2' (s^{-1})	DBV(%)	OEF (%)	CBV	Vessel size(μm)
**patient-01**	Contra-GM	4.72 (3.77)	3.88 (8.50)	39.04 (24.05)	1.94 (2.27)	22.51 (43.39)
	Nonenhancing	4.61 (3.00)	5.99 (8.66)	35.97 (16.82)	1.10 (1.61)	17.47 (22.99)
**patient-02**	Contra-GM	5.58 (6.04)	6.37 (12.48)	43.36 (34.77)	1.63 (1.62)	15.76 (21.60)
	Nonenhancing	2.48 (2.72)	2.53 (7.38)	22.42 (12.47)	0.57 (0.81)	7.86 (7.74)
**patient-03**	Contra-GM	1.72 (3.77)	1.69 (6.29)	17.03 (27.35)	2.28 (1.59)	16.16 (15.00)
	Edema	2.57 (3.04)	3.77 (11.65)	19.63 (13.78)	0.96 (0.70)	18.72 (25.35)
	Enhancing	2.92 (3.82)	3.59 (11.29)	22.47 (20.06)	1.30 (1.52)	69.86 (110.42)
	Necrosis	2.54 (3.34)	3.06 (11.13)	20.32 (18.91)	0.14 (0.55)	18.43 (66.24)
**patient-04**	Contra-GM	4.86 (3.91)	4.29 (8.28)	40.62 (27.31)	0.89 (1.53)	8.34 (18.76)
	Edema	N/A	N/A	N/A	0.19 (0.51)	3.34 (8.77)
	Enhancing	6.41 (9.42)	6.74 (18.28)	45.21 (54.60)	0.72 (1.35)	9.12 (18.96)
	Necrosis	5.32 (9.84)	4.75 (19.71)	43.74 (57.88)	0.09 (0.49)	1.34 (7.31)
**patient-05**	Contra-GM	4.80 (4.34)	4.39 (8.95)	40.09 (27.72)	1.67 (1.23)	14.66 (16.97)
	Nonenhancing	3.09 (2.09)	3.00 (6.02)	27.19 (13.03)	0.56 (0.45)	9.62 (6.94)
	Enhancing	9.44 (9.31)	10.16 (16.11)	68.94 (63.27)	0.84 (0.71)	18.59 (17.34)
**patient-06**	Contra-GM	3.77 (3.68)	3.35 (8.50)	31.23 (21.56)	1.79 (1.88)	16.70 (21.10)
	Nonenhancing	3.25 (2.39)	3.73 (7.48)	26.82 (12.01)	1.56 (1.29)	18.06 (13.64)
	Enhancing	5.90 (6.22)	9.48 (14.60)	43.23 (36.38)	2.60 (1.85)	29.43 (29.93)
**Patient-07**	Contra-GM	6.02 (5.17)	6.25 (11.04)	47.72 (33.86)	1.41 (1.10)	16.88 (19.42)
	Edema	4.40 (3.28)	5.62 (8.06)	34.66 (21.19)	0.51 (0.80)	12.10 (14.09)
	Enhancing	17.09 (23.28)	8.82 (21.67)	145.90 (189.46)	4.87 (3.16)	51.42 (42.44)
**patient-08**	Contra-GM	7.74 (9.86)	3.00 (17.65)	81.01 (90.20)	1.70 (1.62)	18.50 (27.02)
	Edema	5.35 (4.08)	3.00 (5.18)	50.50 (32.45)	0.48 (0.64)	12.07 (11.70)
	Enhancing	11.79 (16.92)	3.65 (17.23)	90.15 (123.33)	3.01 (2.35)	52.95 (53.11)
	Edema	24.30 (25.53)	10.10 (28.95)	189.80 (178.77)	1.55 (2.90)	62.66 (84.01)
**patient-09**	Contra-GM	4.22 (4.83)	3.00 (9.12)	34.16 (32.80)	1.75 (1.98)	19.37 (32.78)
	Edema	N/A	N/A	N/A	0.32 (0.74)	14.18 (25.20)
	Enhancing	11.76 (13.96)	10.52 (22.31)	90.50 (109.21)	1.92 (3.11)	58.83 (111.62)
	Necrosis	10.51 (18.28)	10.19 (20.25)	72.68 (113.18)	0.52 (1.58)	20.85 (83.12)
**patient-10**	Contra-GM	7.96 (7.97)	6.82 (13.32)	64.67 (51.41)	1.46 (1.93)	15.72 (26.87)
	Edema	4.42 (2.45)	3.00 (4.83)	42.27 (16.83)	0.33 (0.78)	9.19 (16.17)
	Enhancing	13.75 (13.65)	11.51 (24.75)	101.78 (92.68)	2.84 (3.10)	44.18 (55.25)
	Necrosis	10.14 (12.06)	8.40 (16.46)	76.32 (80.38)	0.31 (1.19)	4.76 (19.43)

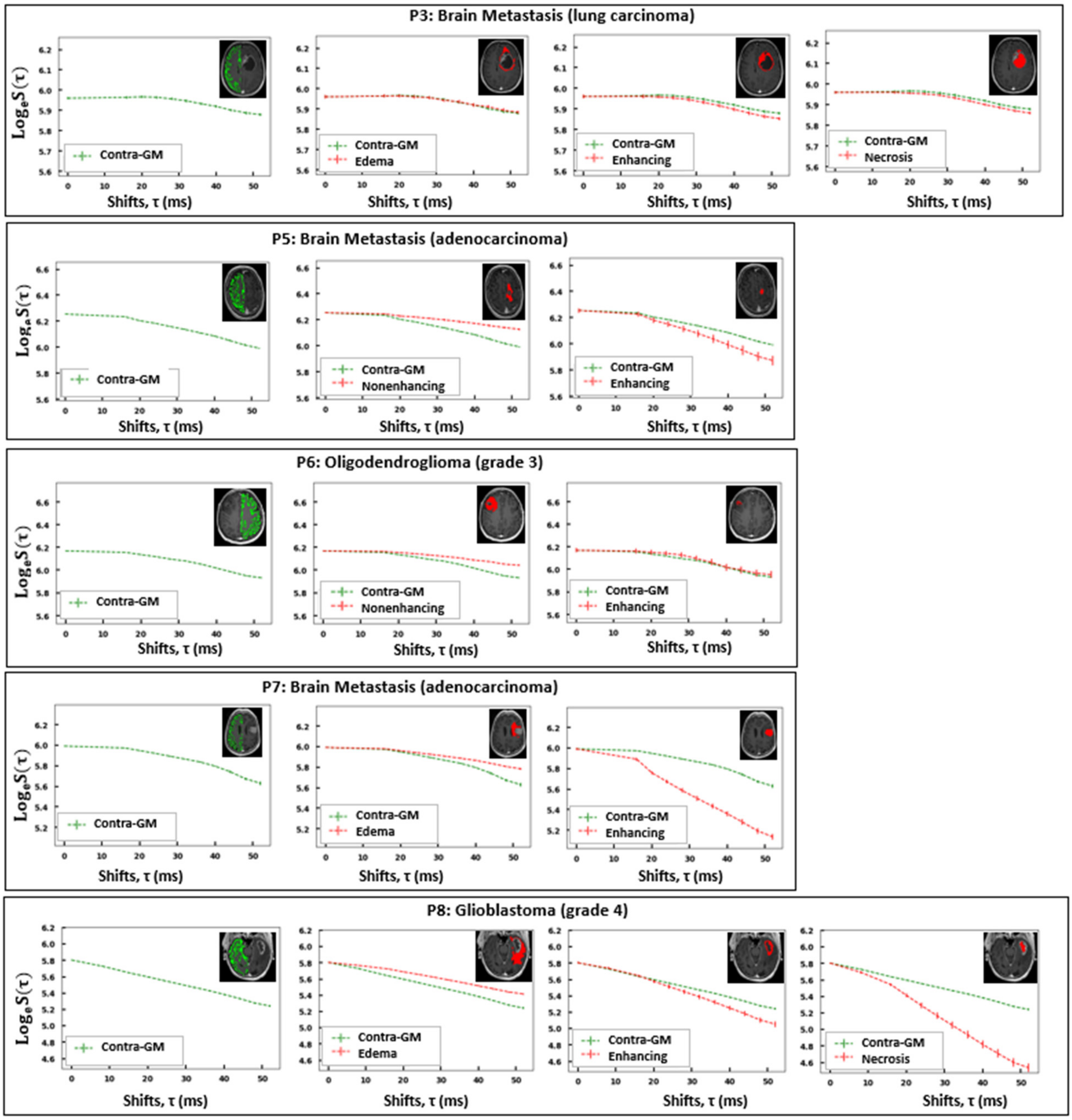

Supplementary Figure S1: The VOI-averaged FLAIR-ASE signal across various VOIs for five patients with brain tumors. The VOIs, are: Contra-GM, Nonenhancing, Edema, Enhancing, and Necrosis. However, not all VOIs are relevant to each tumor type. Error bars represent ± the standard deviation of the signal over the VOI. VOI: volume of interest, Contra-GM: contralateral gray matter, FLAIR-ASE: Fluid attenuated inversion recovery-asymmetric spin echo.

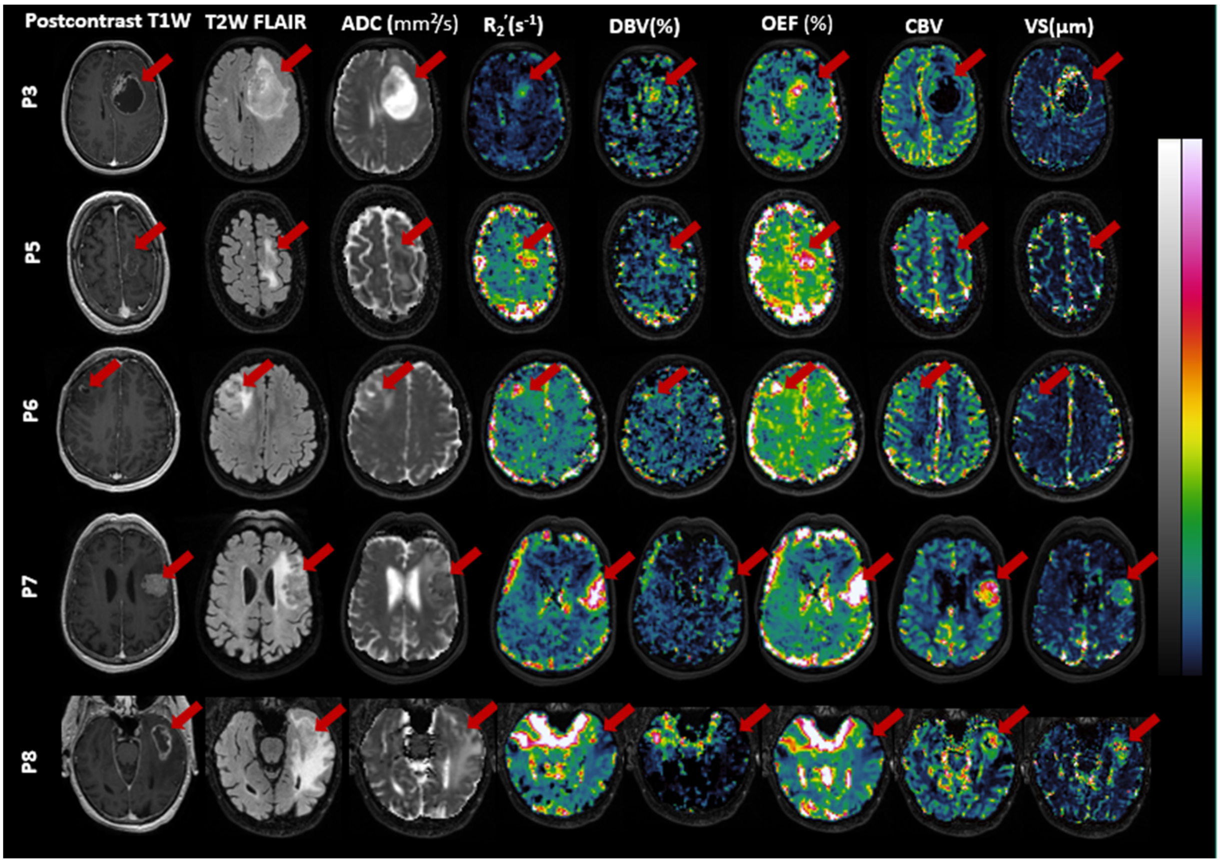

Supplementary Figure S2: Exemplary slices of images including postcontrast T1-weighted (T1W), T2-weighted Fluid-Attenuated Inversion Recovery (T2W FLAIR), Apparent Diffusion Coefficient (ADC), Reversible transverse relaxation rate (R_2'), Deoxygenated Blood Volume (DBV), Oxygen Extraction Fraction (OEF), Cerebral Blood Volume (CBV), and vessel size, are presented, for patients P3 and P5 (Brain Metastasis), P5 (Oligodendroglioma), P7 (Brain Metastasis), and P8 (Glioblastoma). Red arrows highlight the tumors. Intensity scales vary across images, with ADC ranging from 0-3 x 10^{-3} mm²/s, R_2' from 0-20 s^{-1} (0-40 s^{-1} for P7 and P8), DBV from 0-50% (0-100% for P7 and P8), OEF from 0-100% (0-400% for P7 and 0-200% for P8), CBV from 0-10, and vessel size from 0-300 μm. A detailed view of P10's tumor area is included.
